# The evolution of fruit scent: phylogenetic and developmental constraints

**DOI:** 10.1186/s12862-020-01708-2

**Published:** 2020-10-27

**Authors:** Omer Nevo, Kim Valenta, Annemarie Kleiner, Diary Razafimandimby, Juan Antonio James Jeffrey, Colin A. Chapman, Manfred Ayasse

**Affiliations:** 1 Institute of Evolutionary Ecology and Conservation Genomics, Ulm University, Ulm, Germany; 2 German Centre for Integrative Biodiversity Research (iDiv) Halle-Jena-Leipzig, Puschstraße 4, 04103 Leipzig, Germany; 3 Institute of Biodiversity, Friedrich Schiller University Jena, Dornburgerstr 159, 07743 Jena, Germany; 4 Department of Anthropology, University of Florida, Gainesville, FL USA; 5 Faculty of Sciences, Zoology and Animal Biodiversity, University of Antananarivo, Antananarivo, Madagascar; 6 Department of Molecular and Cell Biology, University of Connecticut, Storrs, CT USA; 7 School of Medicine, Case Western Reserve University, Cleveland, OH USA; 8 Department of Anthropology, Center for the Advanced Study of Human Paleobiology, The George Washington University, Washington, DC 20037 USA; 9 School of Life Sciences, University of KwaZulu-Natal, Scottsville, Pietermaritzburg, South Africa; 10 Shaanxi Key Laboratory for Animal Conservation, Northwest University, Xi’an, China

**Keywords:** Developmental constraints, Frugivory, Olfactory communication, Phylogenetic signal, Plant evolution, Seed dispersal

## Abstract

**Background:**

Fruit scent is increasingly recognized as an evolved signal whose function is to attract animal seed dispersers and facilitate plant reproduction. However, like all traits, fruit scent is likely to evolve in response to conflicting selective pressures and various constraints. Two major constraints are (i) phylogenetic constraints, in which traits are inherited from ancestors rather than adapted to current conditions and (ii) developmental constraints, if phenotypes are limited by the expression of other traits within the individual. We tested whether phylogenetic constraints play a role in fruit scent evolution by calculating the phylogenetic signal in ripe fruits of 98 species from three study sites. We then estimated the importance of developmental constraints by examining whether ripe fruits tend to emit compounds that are chemically similar to, and share biosynthetic pathways with, compounds emitted by conspecific unripe fruits from which they develop.

**Results:**

We show that closely related taxa are not more similar to each other than to very distinct taxa, thus indicating that fruit scent shows little phylogenetic signal. At the same time, although ripe and unripe fruits of the same species tend to emit different chemicals, they tend to employ chemicals originating from similar biosynthetic pathways, thus indicating that some developmental constraints determine ripe fruit scent.

**Conclusions:**

Our results highlight the complex landscape in which fruit scent has evolved. On one hand, fruit scent evolution is not limited by common ancestry. On the other hand, the range of chemicals that can be employed in ripe fruits is probably constrained by the needs of unripe fruits.

## Background

Ripe fruits synthesize a wide array of secondary metabolites that fulfill a variety of different functions [1] or potentially no function [2]. Most work on the adaptive functions of these metabolites focused on relatively large and non-volatile compounds with identified functions, such as defense against microbial and invertebrate antagonists [3–5]. In contrast, the study of fruit volatile organic compounds (VOCs)—lightweight secondary metabolites that fruits emit and which are responsible for the tremendous diversity of fruit scents—are few. In recent years there has been growing interest in the ecology and evolution of fruit scent [6]. While it is assumed that at least some fruit VOCs are also involved in fruit defense [7, 8], most of those studies have focused on the roles of fruit scent as an attractant for seed-dispersing vertebrates [6, 9–20].

Most work on the ecological function of fruit scent has focused on the interaction between bats and figs (*Ficus* spp.). Fig scent has been found to function as an attractant to seed dispersing bats in various species in both Paleo- and Neotropics, and bat-dispersed fig species tend to emit qualitatively different [9, 10] and more scent than bird-dispersed species [21]. Moreover, a comparison of various fig species showed that the scent of dispersal-stage syconia changes only in a bat-dispersed species [11], suggesting that scent production is likely to be an evolved signal. Evidence that goes beyond bats and figs and show that fruit scent plays a role in primate-plant interactions has accumulated over the last years [12, 16, 17]. Recently, it has been shown that fruits of species that specialize on seed dispersal by lemurs have evolved to signal ripeness [18] and even nutrient content [22, 23] through scent, and that fruit scent plays a major role in the food acquisition strategies of capuchin monkeys [20]. As such, there is a growing acknowledgment that fruit scent functions as a communication system between plants and seed dispersers.

Yet it is possible that ripe fruit VOC synthesis and emission is predominantly the result of factors unrelated to the interaction with seed dispersers [24]. These may include adaptive responses to other factors like edaphic factors or animal antagonists. They may also include non-adaptive constraints like ancestry, if a trait is inherited rather than adapted to the niche requirements of the species, or developmental constraints, in cases where the expression of a phenotype is at least partially determined by expression of other phenotypes [25, 26]. Other fruit traits, such as size, mass, and nutrient content, have been attributed to common ancestry, rather than selection by animals [27, 28]. Another study quantified the degree of phenotypic integration (a measurement of trait correlation which may indicate constraints in trait evolution) and found that fruit color shows less phenotypic integration than fruit size [29]. Fruit color has been found not to show strong phylogenetic signal [30, 31], and to be limited relative to flowers, which is possibly the result of chemical constraints [31]. Taken together, these studies indicate that in comparison to morphological traits, fruit color may be more malleable to change in response to frugivore behavior, but that its evolution is not fully constraint-free.

Constraints on plant VOC evolution have received less attention, but one study found that plant VOC emissions show less phenotypic integration than insect VOC profiles, and within plants flowers show lower degree of phenotypic integration than leaves [32]. Non-volatile chemical defenses in leaves show little or no phylogenetic signal implying weak phylogenetic constraints [33, 34].

So far, to our knowledge, only two studies addressed whether interspecific variance in fruit scent may be driven primarily by common ancestry. Hodgkison et al. [10] quantified the degree of phylogenetic signal in nine *Ficus* species and reported mixed results: scent profiles of far-related bat-dispersed figs tend to resemble each other more than more closely related bird-dispersed figs, but within the bat-dispersed species closely-related taxa tend to cluster. This indicates that phylogenetic conservatism can manifest differently in different scales, and also that it is more apparent where selective pressures are weaker. More recently, Nevo et al. [18] showed that in a system in Madagascar, ripe fruits of closely-related taxa do not have similar chemical scent bouquets. However, these studies were either confined to a single genus or a single system. Moreover, a major limitation was that they both examined similarity at the compound level. Many fruit VOCs derive from common biosynthetic pathways [35, 36] and are modified only in later stages, often in highly non-specific pathways [37]. Thus, the approach of treating the VOCs that plants emit as independent, and equally likely to evolve in the context of selection, may overestimate the difference between species whose scent profiles are dominated by different VOCs that share a biosynthetic pathway. As a result, phylogenetic constraints may play a role in fruit trait evolution, but it is not clear to what extent they may affect fruit scent.

In addition, fruit scent may be constrained by developmental constraints. Various VOCs are synthesized in both vegetative and reproductive (flower and fruit) plant tissue [19, 38–40]. VOC synthesis and emission in ripe fruits may be limited by the availability of enzyme-encoding genes that are selected primarily for other functions. Moreover, expression patterns in fruits may be driven by upregulation of genes in other plant tissues [2, 41]. Thus, it is possible that ripe fruit scent is not limited only to VOCs selected to be emitted in this stage, but rather a result of VOC synthesis in other parts of the plant.

Here, we examine whether the evolution of fruit scent is subjected to phylogenetic and developmental constraints. We first measure the phylogenetic signal in three independent datasets from Madagascar (Ranomafana National Park—RNP; 30 species), Uganda (Kibale National Park—KNP; 49 species), and Germany (Ulm; 19 species). Since many VOCs derive from common biosynthetic pathways, phylogenetic conservatism can be manifested not only in emission of similar VOCs in closely-related taxa, but also in the emission of biochemically-associated VOCs, i.e., VOCs that share biochemical pathways. We therefore calculate the phylogenetic signal in fruit scent at two levels: the compound and chemical class level, based on the major known biochemical pathways leading to most plant VOCs. To evaluate whether fruit scent is constrained by developmental constraints, we examine whether ripe fruit VOC profile resembles conspecific unripe fruits in a subset of 30 species from one study site, from which we also had data for unripe fruits. Our prediction is that since ripe fruits develop directly from unripe fruits, if developmental constraints dominate ripe fruit scent, it should result in high similarity between the ripe and unripe fruits. Since it has been shown that selective pressures to signal ripeness to animals can render the scent of ripe and unripe fruits different at the compound level in this system [18], we focus on similarity at the chemical class level.

## Results

### Phylogenetic constraints

In all three systems, we found no phylogenetic signal at either the compound or class level, thus indicating that ripe fruit scent is not explained by common ancestry (Table 1; Additional file 1: Fig. S1, Additional file 2: Fig. S2, Additional file 3: Fig. S3).

**Table 1:** (The absence of) phylogenetic signal in ripe fruit scent in three study sites

		K_mult_	p value
RNP, Madagascar	Compound level	0.45	0.22
	Class level	0.49	0.25
KNP, Uganda	Compound level	0.4	0.14
	Class level	0.36	0.58
Ulm, Germany	Compound level	0.37	0.26
	Class level	0.35	0.53

Kmult and p-values are from randomization tests with 1000 permutations. Analysis of K_mult_ in RNP at the compound level reproduces an analysis already reported in Nevo et al. 2018, Science Advances 4: eaat4871. © The Authors, some rights reserved; exclusive licensee American Association for the Advancement of Science. Distributed under a Creative Commons Attribution NonCommercial License 4.0 (CC BY-NC) https://creativecommons.org/licenses/by-nc/4.0/

### Developmental constraints

**Fig. 1:**
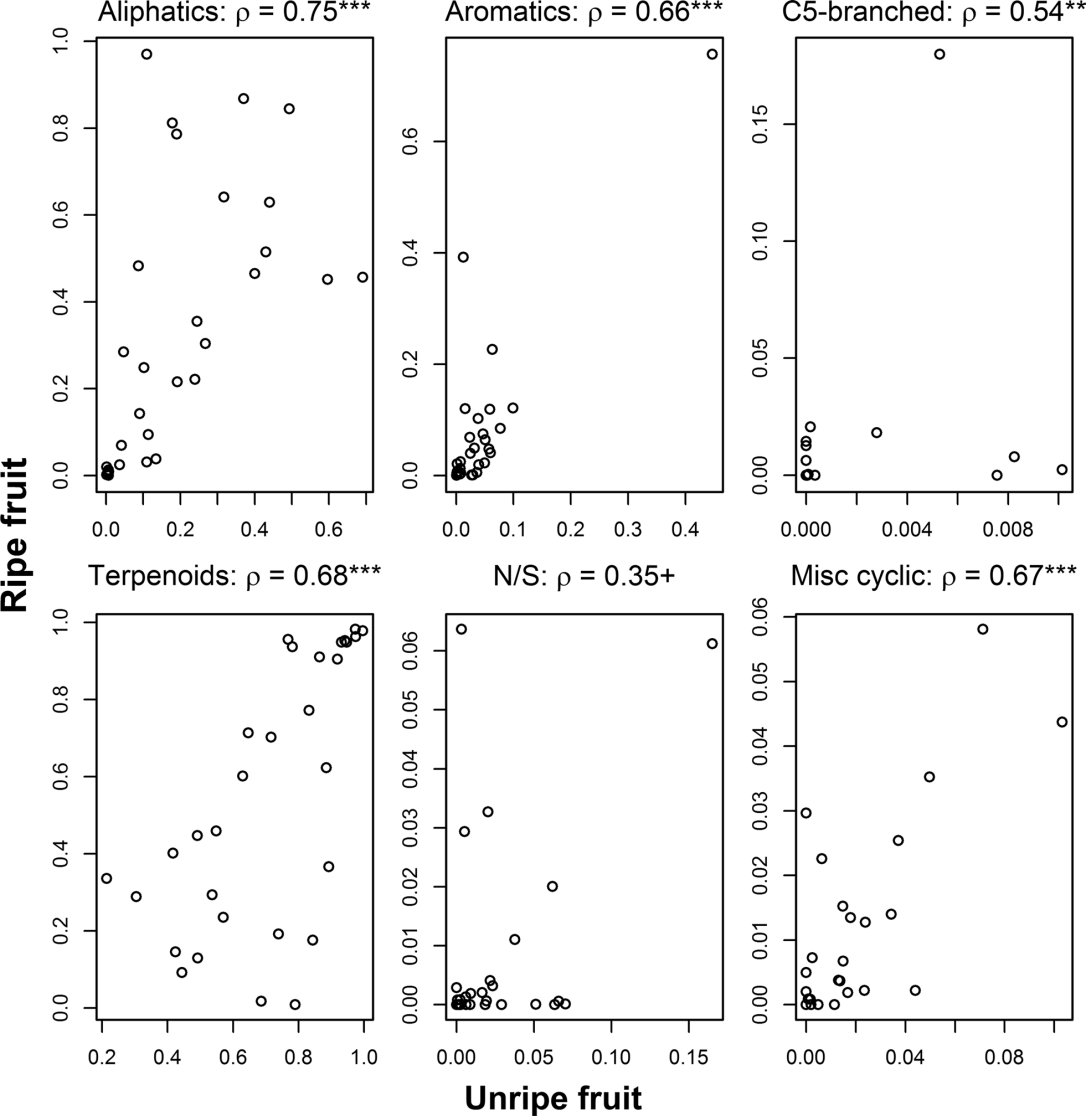
VOC classes in ripe and unripe fruits. Correlation coefficients above the figures are Spearman’s moment correlation coefficient (ρ), which is not affected by outliers and the non-normal distribution. X and Y axes are the relative amount of each VOC class in unripe and ripe fruits. Statistical significance of the correlation coefficients is based on correlation tests. +p < 0.1; **p < 0.01; ***p < 0.001

Ripe fruit scent tended to contain VOCs from the same classes as unripe fruits. We found high positive correlations in the three main chemical classes of plant volatiles: aliphatics (ρ = 0.75, p < 0.001), aromatics (ρ = 0.66, p < 0.001), and terpenoids (ρ = 0.68, p < 0.001), as well as in the less common miscellaneous cyclic compounds (ρ = 0.67, p < 0.001) (Fig. 1). Weaker correlations were found in the much rarer C5-branched (ρ = 0.54, p < 0.01) and nitrogen/sulfur containing compounds (ρ = 0.35, p = 0.06). The weighted average (mean correlation coefficient weighted by the average relative amount of each compound class in ripe fruits) was 0.69.

## Discussion

Our results show that, across three study sites in the tropics and temperate regions, ripe fruit scent does not show any significant trace of phylogenetic signal, i.e. the tendency of closely-related taxa to resemble one another. This is consistent in both compound and chemical class levels. It implies that ripe fruit scent is not strongly constrained by common ancestry and is likely to be more malleable to selective pressures by local disperser groups, pathogens or predators or change as a result of non-adaptive drift. It is remarkable that the phylogenetic signal is not stronger at the chemical class level. The low specificity of latter stage modifying enzymes implies that changes within chemical classes, i.e. at the compound level, should be achieved relatively easily. A change in the compound class would require different biosynthetic machinery which processes different precursors, and was thus predicted to be more conservative.

A possible explanation is that our phylogenetic analysis used a wide group of species from multiple plant families, and indeed multiple origins of fleshy fruits. It is possible that the time since divergence of many clades was sufficient for major shifts in scent production to have occurred. The 98 species covered here are only a minority of fleshy-fruit-producing plants. While a similar analysis on a broader scale may reveal stronger phylogenetic structure, the required data are unlikely to be available soon given the technical difficulties of conducting chemical analysis at such a scale. Indeed, the current study probably represents the largest ever published dataset of fruit scent chemistry. A more productive alternative would be to focus on wider sampling within a narrower phylogenetic clade, in which the effects of phylogeny are more likely to be apparent.

At the same time, while the number of species available for within-genus analysis is too low in our samples, it qualitatively appears that even within more recent divergences ripe fruit chemical profiles do not reflect the phylogeny. For example, five members of families Rutaceae, six members of Rubiaceae, and congeners such as *Cordia* and *Solanum* are more similar to very distant species than to one another (Additional file 1: Fig. S1, Additional file 2: Fig. S2). In contrast, nine *Ficus* species from KNP show stronger conservatism at the class level (Additional file 2: Fig. S2), yet at the same time five *Ficus* species from Madagascar do not (Additional file 1: Fig. S1). Thus, even though the quantitative phylogenetic analysis may be affected by the deep phylogeny of the model systems, the overall pattern is that even within clades phylogeny fails to explain variation in fruit scent, and species are often more similar to far-related taxa in which fleshy fruits have in some cases evolved independently.

In contrast, we found evidence for developmental constraints in ripe fruit scent, as the relative amounts of VOC classes in ripe and unripe fruits showed high positive correlation. Many of the fruit species analyzed here specialize on seed dispersal by lemurs and have been selected to signal ripeness by emitting scent that is significantly different from the scent of unripe fruits [18]. Our results imply that while a shift in the scent profile of a fruit upon ripeness can be achieved by changing the individual VOCs emitted, these compounds tend to belong to the same chemical classes. Thus, it appears that the biochemically easier change in scent profile within chemical class is sufficient to achieve the goal of signaling to vertebrate seed dispersers. The only exception for this rule is aliphatic esters, which tend to be more common in ripe fruits even when absent in unripe fruits [18]. Esters are a product of a downstream process in which products of many of the more basic pathways discussed here are used as substrates. As such, they are likely to represent the best example for ripe fruit VOCs not constrained by unripe fruit chemistry.

Alternatively, it is possible that the high correlations between ripe and unripe fruits at the class level represent adaptation and not constraint. The ecological function of many VOCs is unknown, but it is expected that at least some play a role in fruit defense against predators and pathogens [8], and in cases in which the same function is required in both ripe and unripe fruits, a similarity between them is expected to be under selection. At the same time, in this case we would expect higher similarity at the compound level since it would be cheaper for the plant to simply continue synthesizing the same compounds rather than switch to other chemically-related ones, which is not the case [18]. Thus, a combination of high variability at the compound level and similarity at the class level is in line with a model in which fruits are under selection to change their scent when becoming ripe to signal to frugivores [17, 18], but are constrained to rely on chemicals from the same biosynthetic pathway.

## Conclusions

Our results show that fruit scents of closely related taxa do not tend to be similar and are often more similar to very far-related taxa, thus indicating that there is no or little phylogenetic conservatism in ripe fruit scent. At the same time, within species, the biochemical profile of unripe fruits serves as a blueprint from which ripe fruit scent can diverge, but primarily at the finer compound level. The interplay between weak phylogenetic and noticeable developmental constraints demonstrates a complex adaptive landscape on which fruit scent may evolve: plants can alter their scent relatively easily, but they cannot do so without altering unripe fruit scent as well, which may bring about other costs. These results parallel to some extent the patterns observed for fruit color, which is relatively free of phylogeny [30, 31], but might be constrained by the chemistry of flowers, from which they develop [31]. Application of the approaches we took here on a large sample of closely related taxa, preferably from diverse habitats, may fine-tune the conclusions reached here and shed more light on the evolution of fruit scent.

## Methods

Samples from the three study sites were collected, sampled and analyzed using different protocols and were therefore analyzed separately (Additional file 4: Tables S2–8). All raw data, including species, number of samples per individual and species, and scent data are available in Additional file 4: Tables S1–8.

*Ranomafana National Park, Madagascar (RNP)* 434 ripe and 428 unripe fruits from 90 individuals of 30 species (Additional file 4: Tables S2–3, S6–7) were used. Data are taken from Nevo et al. [18]. See sampling methods and analysis there. Samples were processed and analyzed similarly to Ulm samples (see below).

*Kibale National Park, Uganda (KNP)* We used data published in Nevo et al. 2017 [8]. All samples were collected in January–June 2016. VOCs of between 2 and 10 fruits from each species were sampled within up to 4 h of collection. 285 ripe fruits from 49 individuals of 49 species were used (Additional file 4: Table S4). Fruit VOCs were collected using a dynamic headspace method. We placed fruits in oven bags (large oven bags, Reynold’s™). We used pumps (Gilian 5000, Sensidyne™) to draw air through the bag at a rate of 1 l / min for 4 h. Incoming air was filtered using activated carbon (Sigma Aldrich). Outgoing air containing VOCs was sampled by a sorbent tube containing two VOC traps (Amberlite XAD-2™, 400-200 mg, Sigma-Aldrich). VOCs were extracted from the trap using 3 ml n-hexane (Sigma Aldrich), shaken manually for 5 min.

Following extraction we added to each 190 µl of each sample a 10 µl solution of an internal standard (heptadecane, 200 ng/ml, dissolved in n-hexane). These solutions were then concentrated to 20 µl using a gentle flow of nitrogen. We analyzed the samples by injecting 2 µl to an Agilent 7890B gas chromatograph with a DB-5 column (Agilent; 30 m × 0.25 mm × 0.25 μm), coupled with an Agilent 5977A inert mass spectrometer operating in electron ionization (EI) mode. We used an autoampler to inject the samples splitless onto a cold injection system (Gerstel), which kept the liner at 10 °C. The liner was then heated at 12° C / min to 300° C, a temperature on which it was held for 4 min. Evaporated VOCs were then transferred to the column. Oven temperature was set to 45° C, and helium was used as carrier gas (1 ml / min). The oven program was first held at 45**°** C for 1 min. It then heated at 7**°** C / min to 310**°** C. It was then held at 310° C for 15 min. Transfer line temperature was set to 250**°** C. Analytes in m/z range of 40–300 Da were recorded.

Contaminants were identified based on their presence in control samples (empty bags) sampled in conditions identical to the fruit samples. We also excluded known contaminants (phthalates, siloxanes). Amounts of compounds were quantified using Amdis 2.71, and their identity was determined based on published retention indices and mass spectra (NIST 11 library).

*Ulm, Germany* Fruit samples in Ulm were collected in Oct–Nov 2017 in the forests around the University of Ulm, Ulm, Germany. We selected species opportunistically and included all fleshy fruit species from which we could obtain at least three individuals. We obtained 592 fruits from 88 individuals of 19 species (Additional file 4: Table S5). Fruit samples were brought to the laboratory and processed similarly to the samples from RNP, as described in Nevo et al. 2018 [18]. In short, we enclosed fruits in sampling chambers made of 40 cm oven bags (Toppits) and sampled fruit VOCs using semi-dynamic headspace sampling onto self-made VOC traps containing a mixture of Carbotrap, Tenax and Carbosieve S-III (all Sigma-Aldrich) in equal proportions. We analyzed trapped chemicals using an Agilent gas chromatograph 7890B with an Agilent DB-5 column (30 m × 0.25 mm × 0.25 μm), and a cold injection system (CIS 4C, Gerstel), coupled with an Agilent mass spectrometer 5977A. TDU temperature was initially set to 10° C, and after 1 min climbed at 10° C 15° C / min up to 300° C. It then rested on this temperature for another 15 min. Desorbed VOCs were then transferred to the liner, which was cooled to − 100° C. The liner then started heating at a rate of 12 °C / min up to 290° C, and was kept on this level for 6 min. The sample was then sent to the column, which was set to 50° C. After one minute it began heating at 10° C / min to a max temperature of 325° C, and then kept on this temperature for 20 min more. Temperature of the MS transfer line was 280° C, MS source to 230° C, MS quad to 150° C. MS operated at electron ionization (EI) mode and scanned between 35 and 450 Da.

Similarly to KNP samples, samples were analyzed using Amdis 2.71. We identified VOCs based on their mass spectra using the NIST11 mass spectra library and published retention indices. Known contaminants (e.g. siloxanes, phthalates) were excluded. We also removed all VOCs which were predominantly present in control samples and those which were present in over 90% of the samples in the entire dataset, under the assumption that a VOC present in all species is more likely to be a contaminant than a genuine compound. We further removed very rare compounds: those present only in one species and in less than 25% of the samples; or those which do not constitute more than 1% of the total VOC emission in any of the model species.

### Statistical analysis

#### Phylogenetic signal

Since the three datasets were collected and analyzed using different instruments and procedures, we analyzed each of them separately. In RNP and Ulm, where we had multiple individuals per species, we first calculated the mean amount of each VOC in the species. In all three sites, analyses were conducted on the relative amounts of each VOC or chemical class in a species, which was obtained by dividing their amount by the total amount of VOCs in the species. Compound-level analyses were conducted on the raw data, in which each VOC is a variable. For biochemical class-level analyses, we categorized all VOCs in each system as one of seven chemical classes, based on the biochemical pathway in which they are synthesized [36]: aliphatics (fatty-acid derivatives), aromatics, C5-branched compounds, terpenoids, nitrogen/sulfur containing compounds, miscellaneous cyclic compounds, and unknown (Additional file 4: Table S2–7). We calculated the phylogenetic signal using Kmult [42], following a procedure applied in Nevo et al. 2018 [18]. This method allows calculating a statistic similar to Blomberg’s K [43] in a multivariate dataset. Since our raw data are highly zero inflated and violate the normality assumption of the method, we first calculated the Bray–Curtis dissimilarities between all samples in each of the three datasets. We then ran a principal coordinate analysis (PCoA) to obtain new variables summarizing the distances between all samples, and calculated Kmult on the scores of all species in the PCoA. As such, this approach measures to what degree closely-related species score similarly in the PCoA, (Dean Adams, pers. comm.). We used a phylogeny by Zanne et al. [44]. This phylogeny has some polytomies (unresolved phylogenetic relationships within a genus). However, the presence of polytomies has been shown to inflate type I error, i.e. overestimate the strength of the phylogenetic signal [45]. Since our results all report no phylogenetic signal, the fact that they emerge from analyses slightly prone to false positives strengthens our conclusion that ripe fruit scent does not show strong phylogenetic signal. To visualize the results (Additional file 1: Fig. S1, Additional file 2: Fig. S2, Additional file 3: Fig. S3) we used the same Bray–Curtis dissimilarity matrices and conducted a hierarchical cluster analysis, whose results were compared to the phylogenetic relationships in a tanglegram.

#### Developmental constraints

To examine whether ripe fruit scent is constrained by the VOC profile of unripe fruits, we calculated the Spearman’s moment correlation coefficient in the relative amount of the same VOC classes noted above (aliphatics, aromatics, C5-branched, terpenoids, N/S, miscellaneous cyclic). We used a non-parametric approach because distributions of the variables were not normal and different between VOC classes, and our goal was to obtain a comparable figure for all. We tested the significance of the correlation coefficients (testing whether they are significantly different from 0) using correlation tests. We excluded unknown compounds from this analysis because any relationship between unknown compounds in this analysis would be meaningless. This exclusion is unlikely to affect the results since unknown compounds constitute 0.9% (± 2.1% SD) of the scent profiles of the fruits. We used a non-parametric correlation because many of the distributions were skewed. We further calculated a weighted mean Spearman’s correlation coefficient (average correlation coefficients weighted by the share of ripe fruit scent) to estimate the overall similarity between ripe and unripe fruits. All analyses were conducted on R 3.2.5 [46] using packages ape [47], vegan [48], geomorph [49], phytools [50] and dendextend [51]. R code is available at https://github.com/omernevo/Constraints-in-fruit-scent-evolution.

## Supplementary information

**Additional file 1: Figure S1.** Ripe fruit scent is not explained by phylogeny—RNP (Madagascar). Tangelgram of ripe fruit scent and phylogeny. Left taglegram- compound level; right tanglegram—VOC class level. Center—phylogeny from Zanne et al. 2014. Note that the two middle trees are mirror images of the same tree. Left tangelgram (compound level) is modified from Nevo et al. 2018, Science Advances 4: eaat4871. © The Authors, some rights reserved; exclusive licensee American Association for the Advancement of Science. Distributed under a Creative Commons Attribution NonCommercial License 4.0 (CC BY-NC) https://creativecommons.org/licenses/by-nc/4.0/. Note that the tanglegram is slightly different from the one published there because we used a different algorithm.

**Additional file 2: Figure S2.** Ripe fruit scent is not explained by phylogeny—KNP (Uganda). Tangelgram of ripe fruit scent and phylogeny. Left taglegram- compound level; right tanglegram—VOC class level. Center—phylogeny from Zanne et al. 2014. Note that the two middle trees are mirror images of the same tree.

**Additional file 3: Figure S3.** Ripe fruit scent is not explained by phylogeny—Ulm (Germany). Tangelgram of ripe fruit scent and phylogeny. Left taglegram- compound level; right tanglegram—VOC class level. Center—phylogeny from Zanne et al. 2014. Note that the two middle trees are mirror images of the same tree.

**Additional file 4: Table S1.** VOCs and RIs. **Table S2**. Madagascar—RNP2016. **Table S3**. Madagascar—RNP2017. **Table S4**. Uganda—KNP. **Table S5**. Germany—Ulm. **Table S6**. RNP Unripe 2016. **Table S7**. RNP Unripe 2017. **Table S8**. N individuals per spec.

## Data Availability

All raw data are available in Additional file 4: Table S1-7. R code of all analyses is available at https://github.com/omernevo/Constraints-in-fruit-scent-evolution.

## Abbreviations

VOC: Volatile organic compound
RNP: Ranomafana National Park
KNP: Kibale National Park
EI: Electron ionization
NIST: National Institute of Science and Technology
TDU: Thermal desorption unit
