# Supplementary figures and images for: The evolution of fruit scent: phylogenetic and developmental constraints
Source: BMC Evol Biol. 2020 Oct 27;20:138. doi: 10.1186/s12862-020-01708-2 (PMC7590443; doi:10.1186/s12862-020-01708-2)

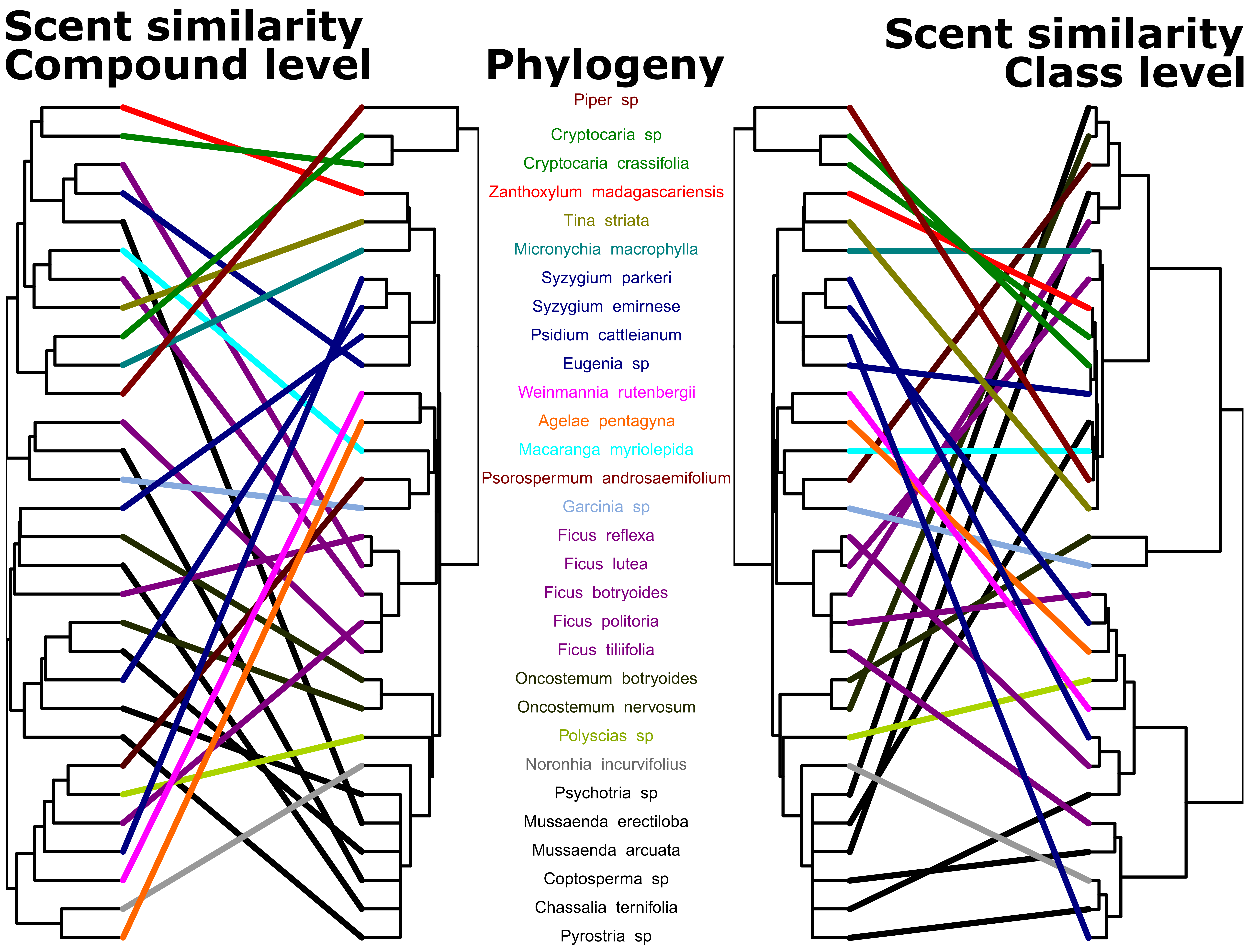

Supplement: Supplementary file 1 — Additional file 1: Figure S1. Ripe fruit scent is not explained by phylogeny—RNP (Madagascar). Tangelgram of ripe fruit scent and phylogeny. Left taglegram- compound level; right tanglegram—VOC class level. Center—phylogeny from Zanne et al. 2014. Note that the two middle trees are mirror images of the same tree. Left tangelgram (compound level) is modified from Nevo et al. 2018, Science Advances 4: eaat4871. © The Authors, some rights reserved; exclusive licensee American Association for the Advancement of Science. Distributed under a Creative Commons Attribution NonCommercial License 4.0 (CC BY-NC) https://creativecommons.org/licenses/by-nc/4.0/. Note that the tanglegram is slightly different from the one published there because we used a different algorithm. [file 12862_2020_1708_MOESM1_ESM.png]

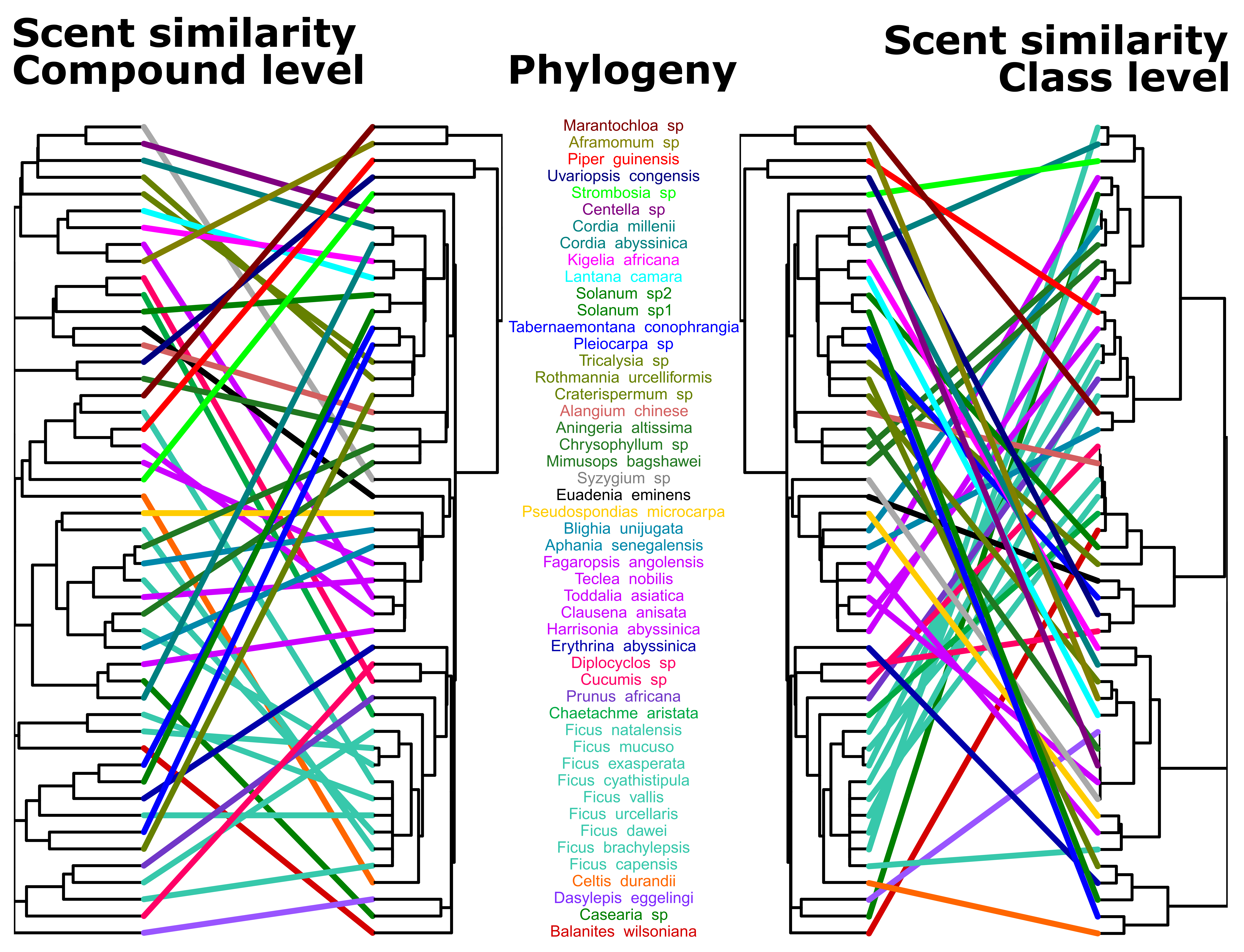

Supplement: Supplementary file 2 — Additional file 2: Figure S2. Ripe fruit scent is not explained by phylogeny—KNP (Uganda). Tangelgram of ripe fruit scent and phylogeny. Left taglegram- compound level; right tanglegram—VOC class level. Center—phylogeny from Zanne et al. 2014. Note that the two middle trees are mirror images of the same tree. [file 12862_2020_1708_MOESM2_ESM.png]

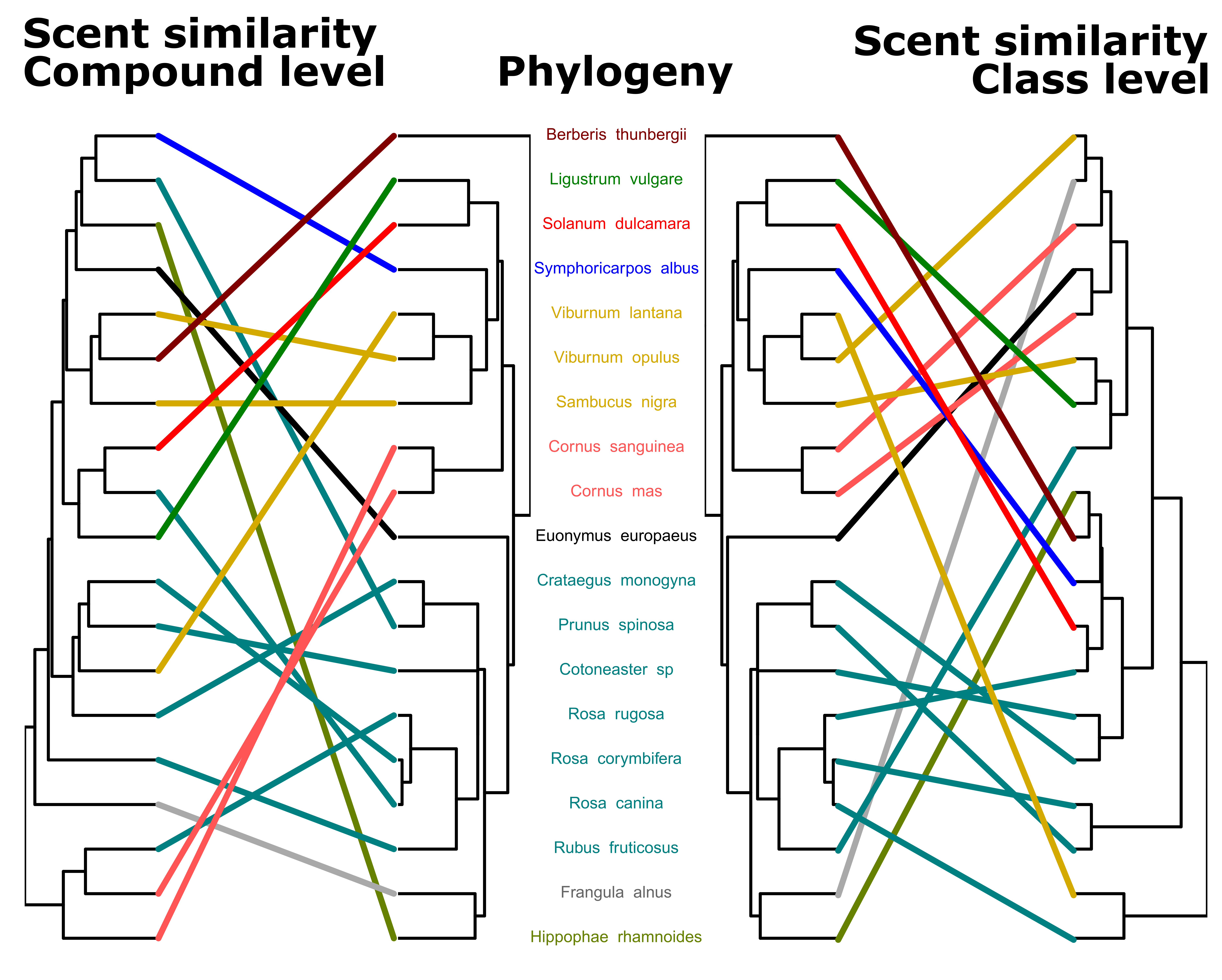

Supplement: Supplementary file 3 — Additional file 3: Figure S3. Ripe fruit scent is not explained by phylogeny—Ulm (Germany). Tangelgram of ripe fruit scent and phylogeny. Left taglegram- compound level; right tanglegram—VOC class level. Center—phylogeny from Zanne et al. 2014. Note that the two middle trees are mirror images of the same tree. [file 12862_2020_1708_MOESM3_ESM.png]
